# A cross-sectional study in adiponectin, glucose metabolism, and body composition in cystic fibrosis

**DOI:** 10.3389/fendo.2024.1382241

**Published:** 2024-10-28

**Authors:** Bibi Uhre Nielsen, Christine Råberg Mikkelsen, Peter Sandor Oturai, Rikke Krogh-Madsen, Terese Lea Katzenstein, Christian Ritz, Tacjana Pressler, Thomas Peter Almdal, Inger Hee Mabuza Mathiesen, Daniel Faurholt-Jepsen

**Affiliations:** ^1^ Cystic Fibrosis Center Copenhagen, Department of Infectious Diseases, Copenhagen University Hospital – Rigshospitalet, Copenhagen, Denmark; ^2^ Department of Clinical Physiology and Nuclear Medicine, Copenhagen University Hospital – Rigshospitalet, Copenhagen, Denmark; ^3^ Centre for Physical Activity Research, Copenhagen University Hospital – Rigshospitalet, Copenhagen, Denmark; ^4^ Department of Infectious Diseases, Copenhagen University Hospital – Hvidovre, Copenhagen, Denmark; ^5^ Department of Nutrition, Exercise and Sports, University of Copenhagen, Frederiksberg, Denmark; ^6^ Department of Endocrinology, Copenhagen University Hospital – Rigshospitalet, Copenhagen, Denmark

**Keywords:** cystic fibrosis, adiponectin, fat mass, muscle mass, bone mass

## Abstract

**Objective:**

We hypothesized that the insulin-sensitizing adipokine adiponectin (ADP) is upregulated in cystic fibrosis (CF) related diabetes (CFRD) and underweight adults with CF. We aimed to assess correlations between glucose metabolism, body composition and ADP in CF.

**Methods:**

We performed a cross-sectional study among adults with CF at the Copenhagen CF Center. The study included a fasting level of ADP, an oral glucose tolerance test (OGTT), and a dual energy-x-ray absorptiometry scan.

**Results:**

In total, 115 patients were included of whom 104 had an OGTT performed. Glucose intolerance was not correlated with ADP in multivariable analysis, while increased hepatic insulin resistance (i.e., HOMA-IR) was correlated with reduced ADP levels. ADP declined by 4% (e^β^ 0.96, 95% CI: 0.94, 0.98), 5% (e^β^ 0.95, 95% CI: 0.93, 0.98), 9% (e^β^ 0.91, 95% CI: 0.87, 0.95), and 83% (e^β^ 0.17, 95% CI: 0.08, 0.37) for each one unit (kg/m^2^) increase in body mass index, fat mass index, muscle mass index, and bone mineral content index, respectively.

**Conclusions:**

In CF, ADP was negatively correlated with hepatic insulin resistance as well as low fat, muscle, and bone mass, but not with glucose intolerance. This suggests that malnutrition leads to higher ADP levels in CF.

## Introduction

The fat tissue hormone adiponectin (ADP) is predominantly produced by adipocytes and is considered an important link between fat metabolism and glucose regulation (1, 2). ADP regulates the energy homeostasis, through insulin-sensitizing effects (3), upregulation of the expenditure of stored energy and improvement of the lipid metabolism (4), and this seem to affect both the body composition and the glucose metabolism (5). Consistently, ADP was found to be negatively correlated with fat mass and fasting glucose in obese individuals and thus a higher level of ADP is thought to be related to improved glucose metabolism in healthy normal-weight adults (5).

ADP improves insulin secretion and insulin sensitivity (4, 6), meanwhile insulin is a negative regulator of ADP (7, 8). Accordingly, enhanced levels of ADP have been shown in several studies in type 1 diabetes (9, 10). This suggests that increased ADP levels seen in type 1 diabetes is a compensatory mechanism aiming at stimulating beta cell function (9), and a similar compensation may be observed in other types of pancreatogenic diabetes.

Cystic fibrosis (CF) related diabetes (CFRD) is a type of pancreatogenic diabetes characterized by decreased insulin secretion as well as low body mass index (BMI) explained by both low muscle and low fat mass (11). Hence, although type 1 diabetes and CFRD have very different pathophysiology, pancreatic insulin deficiency and hyperglycemia are present in both diseases. Therefore, ADP may also be increased in CFRD, and, in theory, elevated ADP could postpone onset of CFRD by improving both insulin secretion and insulin sensitivity. However, existing data do not fully support this theory. In two studies among adults with CF, Ziai et al. found no clear association between ADP and the total insulin secretion (12) and Hammana et al. reported no association between insulin resistance and ADP among individuals with CF (13). Thus, ADP levels might be influenced by other factors than glucose tolerance in individuals with CF.

We recently reported that individuals with CF and glucose intolerance had reduced BMI (14). Therefore, we hypothesized that the reduction in body mass can confound a potential correlation between ADP and glucose intolerance. This is further supported by Panagopoulou et al., who found that ADP was increased in individuals with a low fat free mass in a study including both individuals with and without CF (11). However, this finding might be biased, as individuals with CF seem to have higher ADP levels compared to non-CF controls (15, 16). Furthermore, no studies have shown that fat mass is negatively correlated with ADP levels in CF.

To our knowledge, existing data in CF populations have related ADP to glucose metabolism and body composition in separate studies. Hence, this supports studying ADP in relation to both glucose metabolism and body composition in the same CF population to expand the current insight in the ADP metabolism in CF. We therefore aimed to assess the association between glucose intolerance, insulin resistance and ADP as well as the associations between measures of body composition (i.e., fat mass, muscle mass, bone mass) and ADP among adults with CF in the Copenhagen CF cohort.

## Methods

This study on ADP was nested in a cross-sectional study including 134 adults (≥18 years) with CF conducted at the Copenhagen CF center in 2017 prior to the new CF modulator treatment [for details (14)]. The study was approved by the Danish Ethical Committee (H-16022305) and all participants signed the consent after receiving oral and written information about the study. Demographic data, spirometry, and exocrine pancreas function data were obtained from the Danish CF database.

ADP and glucose tolerance were obtained during a standard 2-hour oral glucose tolerance test (OGTT) done during routine visits with clinically stable participants. The participants were fasted for 8 hours before the OGTT. The OGTT included venous blood (i.e., EDTA) samples collected prior, and 1 and 2 hours after intake of 75 g anhydrous glucose. The samples were centrifuged and frozen (-80°C) within 1 hour for the purpose of analyzing all samples in one batch. Fasting plasma (p) was used to assess total ADP levels quantified by duplicate enzyme linked immunosorbent assay analyses (Biovendor, Karasek, Czech Republic). P-insulin (e801 module) and p-glucose (c702 module) were analyzed using a Cobas 8000. Normal glucose tolerance status (NGT), indeterminate glucose tolerance, impaired glucose tolerance (IGT), and CFRD were classified according to the American Diabetes Association CFRD Consensus Statement (17). Homeostatic model assessment for insulin resistance (HOMA-IR) was used as a marker for hepatic insulin resistance (18, 19). Matsuda index equation was used to calculate peripheral insulin sensitivity from the three time points of the OGTT (20, 21), and the 1-hour insulin secretion rate (ISR_1hour_) was used as a marker of beta cell function (22). At the day of the OGTT, the C-reactive protein (CRP) (normal range: <10 mg/L) were measured (Cobas 8000, c702 module) as a marker of systemic inflammation.

Body composition was assessed by a dual energy X‐ray absorptiometry (DXA) scanner (Lunar Prodigy Pro, GE Healthcare, Madison, WI, USA). The estimates included total fat mass, muscle mass, and bone mass (bone mineral content). Equivalent to body mass index (BMI, kg/m^2^), all measurements (mass in kilo) were divided with squared height (m^2^) to obtain fat-, muscle-, and bone mineral content indices. Weight and height were measured on the day of the DXA scan.

### Statistical analyses

Data were summarized as either medians (interquartile range, IQR) or counts (percentage). To assess the correlations between the measurements of glucose metabolism (glucose tolerance and insulin resistance) and ADP, linear regression models adjusted for age and sex (minimal adjusted models) and additionally adjusted for exocrine pancreas function (sufficient/insufficient), CRP, and fat mass index (multivariable models) were fitted. We used linear regression models adjusted for age and sex (minimal adjusted models) as well as linear regression models additionally adjusted for exocrine pancreas function (sufficient/insufficient) and CRP (multivariable models) to assess the correlations between measurements of body mass compartments and ADP levels. In all analyses, ADP was logarithmic transformed, and β-coefficients from the linear models were subsequently back transformed to derive the correlations as ratios. To convert the correlations to percentage reduction or increase per one unit increase of the independent variable, 1 was subtracted from the ratios and multiplied by 100. Sensitivity analyses including both fat mass index and muscle mass index or bone mineral content index were also carried out to adjust for potential confounding. Data were analyzed with R x64.

## Results

The study included 115 participants, among whom all had fasted blood sampling and a DXA scan conducted and 104 (90%) also consented to an OGTT. **Table 1** presents the clinical characteristics with an almost equal number of study participants in each of the four glucose tolerance groups (i.e., NGT, indeterminate glucose tolerance, IGT, and CFRD). The median [IQR] ADP level was 10.0 μg/mL [6.3-12.0 μg/mL].

**Table 1:** Background characteristics of 115 adults with cystic fibrosis.

Demographics	
Age	33.3 (25.4, 40.8)
Female	49 (43%)
Mutation	
Homozygous F508-del	71 (62%)
Heterozygous F508-del	41 (36%)
Other	3 (3%)
Lung disease	
FEV1%	74.5 (50.3, 92.4)
Chronic pulmonary infection	84 (73%)
Lung transplantation	15 (13%)
Inflammation status	
C-reactive protein (mg/L)	2.0 (1.0, 6.0)
Oral corticosteroids^a^	18 (16%)
Pancreas disease	
Pancreas insufficient	100 (87%)
Body composition	
Body mass index (kg/m^2^)	22.5 (20.1, 24.6)
Fat mass index (kg/m^2^)	5.2 (3.7, 7.5)
Muscle mass index (kg/m^2^)	16.0 (14.7, 17.5)
Bone mineral content index (kg/m^2^)	0.9 (0.8, 0.9)
Glucose tolerance	
Normal glucose tolerance	32/101 (32%)
Indeterminate	19/101 (19%)
Impaired glucose tolerance	22/101 (22%)
Cystic fibrosis-related diabetes	28/101 (28%)

Data shown as median (IQR) or count (percentage).

Missing data: 104/115 underwent an OGTT; 101 had 1-hour glucose and 101 had 2-hour glucose during the OGTT.

a Prednisolone was the only oral corticosteroid prescribed with the doses ranging between 2.5-12.5 mg. Among those treated with oral corticosteroids, seven had no OGTT conducted, one presented with normal glucose tolerance, five with impaired glucose tolerance and six with cystic fibrosis-related diabetes. The group treated with oral corticosteroids had 10% (95% confidence interval; 2.6-17.1%) lower body mass index compared to the group not receiving treatment.

FEV1%; forced expiratory volume in one second percent predicted.

**Figure 1:**
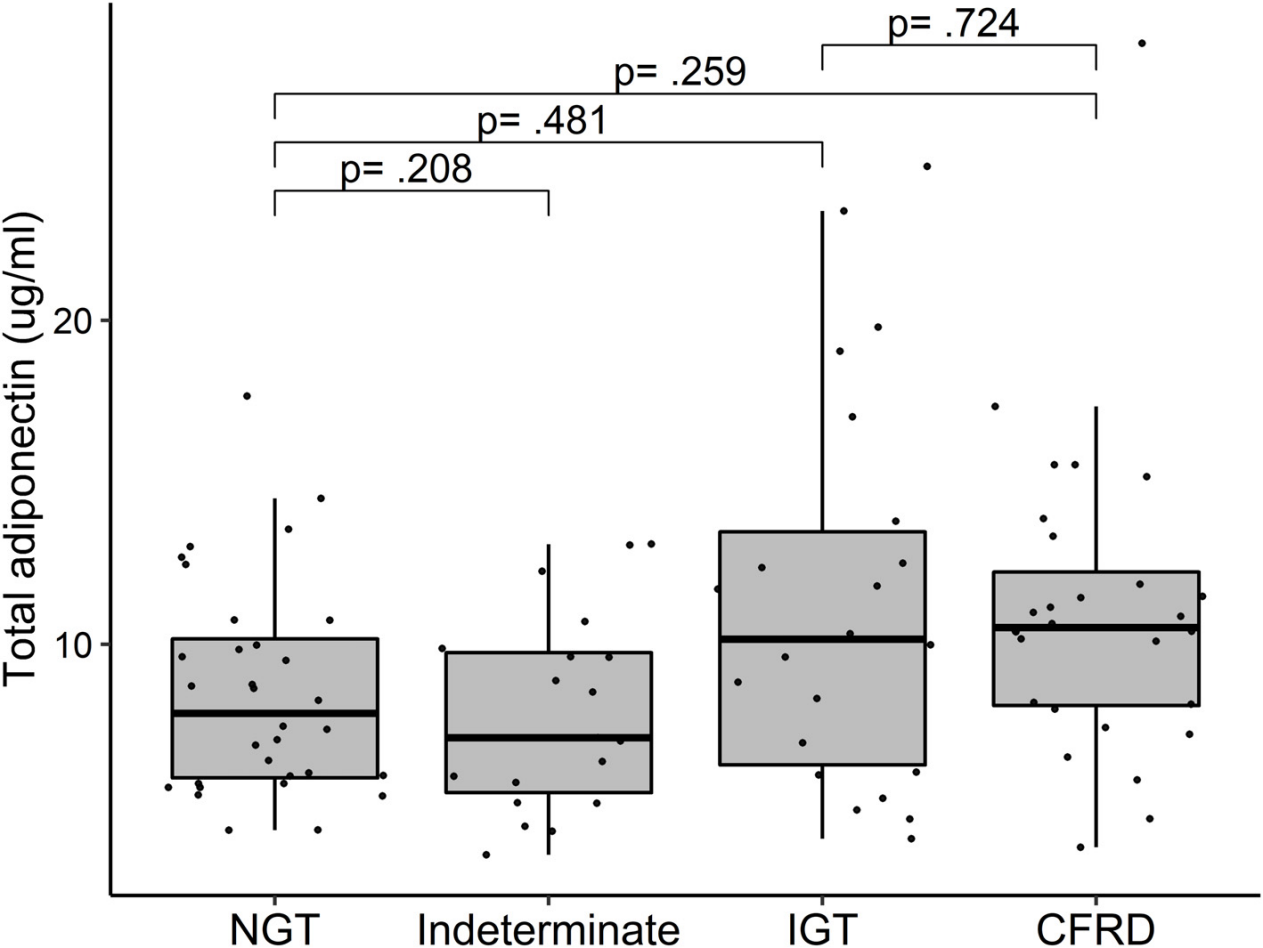
Adiponectin levels among 101 adults with cystic fibrosis stratified by glucose tolerance. P-values were calculated in a linear regression model with logarithmic transformed adiponectin and adjusted for age, sex, exocrine pancreas function (sufficient/insufficient), C-reactive protein and fat mass index. Missing data: Among 104 with an OGTT, glucose tolerance was assessed in 101. NGT, normal glucose tolerance; IGT, impaired glucose tolerance; CFRD, cystic fibrosis related diabetes.

In models adjusted for age and sex, those with IGT had borderline higher ADP levels compared to NGT (e^β^ 1.21, 95% CI: 0.98; 1.59, p=0.078) and those with CFRD had higher ADP levels compared to NGT (e^β^ 1.25, 95% CI: 1.03; 1.53, p=0.026). However, in the fully adjusted models including exocrine pancreas insufficiency, CRP and fat mass index as covariates the estimates for IGT vs NGT (e^β^ 1.09, 95% CI: 0.85; 1.41) and CFRD vs NGT (e^β^ 1.14, 95% CI: 0.91; 1.44) were lower. In multivariable analyses, we found no correlation between glucose tolerance groups and ADP (**Figure 1**). We found no correlation between p-glucose and ADP, while higher levels of p-insulin were correlated with lower levels of ADP, with the strongest correlation seen between fasting p-insulin and ADP (**Table 2**). Further, insulin sensitivity (inverse HOMA-IR and Matsuda index) was correlated with higher levels of ADP, while we found no correlation between insulin secretion (ISR_1hour_) and ADP levels (**Table 2**).

**Table 2:** Linear regression models between measurements of glucose metabolism and adiponectin in 115 individuals with cystic fibrosis.

	N	Adjusted for age and sex				Multivariable			
		e^β^	95% CI		p-value	e^β^	95% CI		p-value
Plasma glucose (mmol/L)									
Fasting	115	1.00	0.97	1.04	.830	1.00	0.96	1.04	.955
1-hour	101	1.00	0.99	1.02	.576	1.00	0.98	1.02	.856
2-hour	101	1.01	1.00	1.02	.057	1.01	1.00	1.02	.170
Plasma insulin (pmol/L/100)									
Fasting	115	0.54	0.40	0.74	<.001	0.62	0.43	0.89	.010
1-hour	101	0.94	0.91	0.98	.002	0.96	0.92	1.01	.095
2-hour	101	0.95	0.92	0.99	.009	0.97	0.93	1.02	.215
Indices									
HOMA-IR	115	0.85	0.78	0.93	<.001	0.88	0.81	0.97	.009
Matsuda index	98	1.01	1.00	1.03	.032	1.01	1.00	1.02	.192
ISR_1hour_	101	0.95	0.85	1.05	.291	1.00	0.87	1.14	.944

Data are based on back transformed β-coefficients from linear regression models with logarithmic transformed adiponectin. Minimal adjusted analyses were adjusted for age and sex and multivariable analyses were adjusted for age, sex, exocrine pancreas function (sufficient/insufficient), C-reactive protein and fat mass index.

Missing data: 104/115 underwent an OGTT; 101 had 1-hour glucose and 101 had 2-hour glucose during the OGTT.

HOMA-IR, Homeostatic Model Assessment of Insulin Resistance; ISR_1hour_, Insulin secretion rate.

BMI, fat mass index, muscle mass index, and bone mineral content index were all negatively correlated with ADP in both minimal adjusted analyses and multivariable analyses indicating that for each one kg/m^2^ increase in BMI, fat mass index, and muscle mass index the levels of ADP decreased by 4-9% (p<0.05, **Table 3**). In multivariable sensitivity analyses including both fat mass index and muscle mass index, the negative correlation between fat mass index and ADP persisted although it was no longer statistically significant; ADP was 3% lower (e^β^ 0.97/kg/m^2^, 95% CI: 0.94; 1.00, p=0.078) for each one unit (kg/m^2^) increase in fat mass index. The correlations between muscle mass index and ADP (e^β^ 0.93/kg/m^2^, 95% CI: 0.88; 0.97, p<0.001) and between bone mineral content index and ADP (e^β^ 0.23/kg/m^2^, 95% CI: 0.10; 0.53, p<0.001) were not affected by adjustment with fat mass index.

**Table 3:** Linear regression models between measurements of body composition and adiponectin in 115 adults with cystic fibrosis.

	N	Adjusted for age and sex				Multivariable			
		e^β^	95% CI		p-value	e^β^	95% CI		p-value
Body composition									
Body mass index (kg/m^2^)	115	0.96	0.94	0.98	<.001	0.96	0.94	0.98	<.001
Fat mass index (kg/m^2^)	115	0.96	0.93	0.98	.002	0.95	0.93	0.98	.002
Muscle mass index (kg/m^2^)	115	0.91	0.87	0.95	<.001	0.91	0.87	0.95	<.001
Bone mineral content index (kg/m^2^)	115	0.16	0.08	0.34	<.001	0.17	0.08	0.37	<.001

Data are based on back transformed β-coefficients from linear regression models with logarithmic transformed adiponectin. Minimal adjusted analyses were adjusted for age and sex and multivariable analyses were adjusted for age, sex, exocrine pancreas function (sufficient/insufficient) and C-reactive protein.

## Discussion

In this study among adults with CF, we found that ADP levels were unrelated to p-glucose and insulin secretion rate, while ADP was inversely correlated with p-insulin levels and positively correlated with insulin sensitivity. Moreover, ADP was negatively correlated with BMI, fat mass, muscle mass, and bone mass.

Overall, severe glucose intolerance was associated with higher ADP levels, although this was partially confounded by pancreas insufficiency, inflammation, and fat mass index. Hence, the association was likely explained by fat mass rather than glucose intolerance. This is similar to previous studies in CF showing that ADP did not change with glucose tolerance status (12, 13). Likewise, we did not find any correlation between insulin secretion rate and ADP, which is also comparable to a former study in individuals with CF (12). However, we did see that the measured p-insulin levels and markers of insulin sensitivity were correlated with ADP and not affected by adjustment with fat mass. HOMA-IR, usually considered a marker of hepatic insulin resistance (23), seemed to be stronger linked to levels of ADP compared to the Matsuda index, which is a better marker of peripheral insulin sensitivity (24). Yet, CF liver disease does not seem to explain the inverse correlation between hepatic insulin resistance and ADP, as CF liver disease has been linked to elevated levels of ADP in CF (11). In other words, ADP is unlikely to explain the link between CF liver disease and glucose intolerance (25) as well as the relationship between reduced insulin sensitivity and CFRD (26). This indicates that CFRD should be differentiated from non-alcoholic fatty liver disease and type 2 diabetes and treatments targeting these diseases should therefore not be used as CFRD management in lean individuals without thorough evaluation.

We also found that all measures and compartments of body mass were negatively correlated with ADP, which is in line with a recent CF study from Qatar (27), but in contrast with a previous CF study from Canada, which reported no correlation between BMI and ADP (13). However, it is not clear from the previous study whether the model was adjusted for sex and age, which is important, since cofactors such as female sex (13) and increasing age (28) are correlated with increased levels of ADP. Further, we showed that fat mass index was negatively correlated with ADP, which seems to be in contrast to a study by Panagopoulou et al., who found a positive association between body fat percentage and ADP (11). However, their study included non-CF controls, thus our study design is not comparable to their study design and differences should be interpreted with caution. Although our findings deviated from previous studies in CF, we found similar trends as is known from several non-CF studies reporting negative associations between fat mass and ADP (29–31). Furthermore, we found increased muscle mass to be correlated with low ADP, unrelated to the fat mass. This is in line with data from Polito et al., who demonstrated that frequent physical exercise lead to lower levels of ADP (15). Similarly, in a non-CF study among individuals with a high prevalence of sarcopenia, both muscle mass index and muscle strength were negatively correlated with ADP (32). This highlights that though ADP levels are strongly connected to body composition and most likely plays important regulatory roles in CF metabolism, ADP appears to be less valuable for evaluating nutritional status in clinical settings.

Following the introduction of new highly effective modulator therapy in the CF population, real-life studies have found significant increases in body mass (33). This change is likely to lead to a reduction in ADP, potentially worsening hepatic insulin sensitivity. Although, hepatic insulin sensitivity does not appear to be the cause of CFRD, it could further complicate disease severity and management. This suggests that incretin-based therapies may play a role in CFRD treatment in near future, as this class of drug seems to enhance ADP levels during treatment (34). However, such interventions should be watchfully introduced, as the current literature provides little knowledge about what role adiponectin plays in pulmonary inflammation and lung function deterioration, and increasing ADP may therefore have both protective (35) or harmful effects on lung function (27).

Finally, we did see that lower bone mass was correlated with higher ADP levels. Although CF bone disease and osteoporosis are common and the results mimic data from individuals with anorexia nervosa, where osteoporosis was linked to higher ADP levels (36), these data should be interpreted with caution. If any true association do exist between bone mass and ADP, we speculate that ADP could stimulate osteoclast formation and thus reduce bone mass, as has been seen in *in vitro* studies (37). Even so, as individuals with CF are often malnourished, reduced fat and muscle mass might lead to increased ADP as well as a subsequent loss of bone mass and thus correlations between bone and ADP might be a chance finding due to the impact from other body compartments, which strongly correlate with bone mass.

As ADP may have a role as an anti-inflammatory agent (3), chronic inflammation could have been correlated with lower levels of ADP in CF (16). However, we found no impact from inflammation in our study as adjusting for CRP did not affect the correlations between body mass and ADP, and the CRP was generally low, and participants were clinically stable without signs of pulmonary exacerbations. At the same time, a limitation in this study is that we only measured total ADP without assessing the high molecular weight isoform, a sub-component of ADP suggested to be a superior marker of insulin sensitivity (38). Moreover, despite the adjustment for fat mass, the results could still be partly confounded by the effect of fat mass, as fasting insulin, HOMA-IR, muscle, and bone mass are all positively correlated with fat mass and difficult to separate completely. Lastly, as this was a cross-sectional study, all mentioning of causal relations are speculative.

## Conclusions

Among adults with CF, we found no correlation between glucose tolerance and ADP, although insulin sensitivity was correlated with higher levels of ADP. Moreover, we found that all measures of body composition were negatively correlated with ADP similar to non-CF populations. This suggests that malnutrition irrespective of CF is associated with higher ADP levels and with increasing body weight after the new highly effective modulator therapy, factors such as fat mass, ADP levels and hepatic insulin sensitivity may become more important in the development of glucose intolerance in CF.

## Data Availability

Qualified researchers can request access to patient-level data after publication, including analytic code. Patient-level data will be anonymized to protect the privacy of the participants. Proposals should be directed to the corresponding author to gain access. Requests can be made after publication and until 5 years following article publication. Requests to access the datasets should be directed to Bibi Uhre Nielsen, bibi.uhre.nielsen.01@regionh.dk.
